# Acupuncture combined with Chinese herbs versus Chinese herbs alone to improve the clinical efficacy of uterine fibroids: a systematic review and meta-analysis

**DOI:** 10.3389/fonc.2024.1456809

**Published:** 2024-11-21

**Authors:** Tianyu Chen, Xin Chen, Wei He, Xiaojing Ma, Zheng Zuo

**Affiliations:** College of Acupuncture and Tuina, Yunnan University of Traditional Chinese Medicine, Yunnan, China

**Keywords:** acupuncture, Chinese herbal medicine, uterine fibroids, clinical efficacy, systematic review, meta-analysis

## Abstract

**Objective:**

The objective of this study was to evaluate the clinical efficacy of acupuncture combined with Chinese herbs versus Chinese herbs alone in treating uterine fibroids.

**Methods:**

A literature search of eight databases identified nine randomized controlled trials (RCTs) evaluating the clinical efficacy of acupuncture combined with Chinese herbs compared to Chinese herbs alone for treating uterine fibroids. Subsequently, data extraction and analysis were conducted to assess the methodological quality and risk of bias in the studies, followed by an analysis of the data from the randomized controlled trials.

**Results:**

Nine randomized controlled trials involving 640 women were included. The results indicated that acupuncture combined with Chinese herbs significantly increased the overall clinical efficacy rate [Z=5.00, P=0.74, relative risk(RR)1.20, 95%CI 1.12 to 1.30, P<0.00001, I²=0%] and reduced the size of uterine fibroids [Z=2.95, P=0.003, SMD=-0.82, 95%CI -1.36 to -0.27, P<0.00001, I²=90%].

**Conclusions:**

Studies have shown that acupuncture combined with Chinese herbs reduces uterine size, lowers hormone levels, and improves quality of life. According to the findings of this study, acupuncture combined with Chinese herbs has a more positive effect on the treatment of uterine fibroids than Chinese herbs alone. However, due to the limited number and quality of the included studies, these conclusions need to be validated by further high-quality research.

**Systematic review registration:**

https://www.crd.york.ac.uk/PROSPERO/#myprospero, identifier CRD42024507248.

## Introduction

Uterine fibroids (uterine smooth muscle tumours, fibroids, or uterine fibroma) (1) are the most common type of benign tumour in the female reproductive system (2). Several factors can trigger the development of fibroids, with the primary ones being race, increasing age, family history, obesity, number of births, smoking, and lifestyle (2–5). Uterine fibroids can occur at various ages, but in China, they are most common in women between the ages of 30 and 50, with the highest incidence between 40 and 50 years old. Approximately 66% of women at the age of 50 are affected by the condition (6). Statistically, the cumulative incidence of uterine fibroids by the age of 50 is as high as 80% in African-American women and about 50% in white women (7). However, the condition is rare in women under 30, and it is extremely uncommon in women under 20. The incidence of uterine fibroids continues to rise with changes in lifestyle habits.

Most patients with uterine fibroids do not exhibit clinical symptoms (8) and require pelvic or ultrasound examinations to confirm the diagnosis, as well as to assess the location and size of the fibroids (9). However, some patients may experience symptoms such as abnormal uterine bleeding, pelvic pressure, bowel dysfunction, or genitourinary issues (10–12), which can severely affect their quality of life and daily activities. Currently, the most commonly used treatments are surgery and medication. Surgical options include myomectomy, laparoscopic dissection, uterine artery embolization, and magnetic resonance-guided focused ultrasound surgery, among others (13–15). However, these procedures often result in unavoidable side effects such as pain, fever, fertility complications, and a high likelihood of recurrence (16, 17). Pharmacological treatments include high-dose progesterone injections, oral contraceptives, and mifepristone (18), but long-term use of these medications can lead to estrogen deficiency. Both surgical and pharmacological treatments consume significant social and medical resources, placing a heavy burden on individuals and the healthcare system.

raditional Chinese Medicine (TCM) is becoming increasingly popular worldwide as a complementary and alternative treatment for uterine fibroids, with acupuncture being used to treat the condition by stimulating specific acupuncture points (19). Various methods can be used to stimulate these points, including acupuncture, electroacupuncture, warming needles, transcutaneous electrical nerve stimulation (TENS), acupoint injections, and moxibustion. Acupuncture can regulate pelvic nerves, balance female sex hormone levels, and improve blood circulation (20). Numerous clinical and animal studies have demonstrated that acupuncture can help alleviate symptoms such as heavy menstruation (21), abdominal pain, and lower back pain, as well as reduce the size of fibroids (22) in patients with uterine leiomyomas. Additionally, high-frequency TENS has been shown to reduce blood flow to the ovaries in rats (23). Other studies have indicated that using TCM or Western medicine alone has limited effectiveness in treating uterine fibroids due to the presence of numerous complications (24). Therefore, acupuncture is a reasonable alternative treatment for fibroids.

There are no detailed, high-quality, systematic methodological evaluations on the treatment of uterine fibroids using acupuncture combined with Chinese herbs. Therefore, the main objective of this study was to assess the efficacy and safety of acupuncture combined with Chinese herbs in improving the symptoms of patients with uterine fibroids. To achieve this, a systematic review and meta-analysis were conducted to compare the efficacy of acupuncture combined with Chinese herbs versus Chinese herbs alone in alleviating the symptoms of uterine fibroids, providing new evidence for the use of acupuncture combined with Chinese herbs as a treatment for uterine fibroids.

## Materials and methods

This study was conducted under the Preferred Reporting Items for Systematic Evaluation and Meta-Analysis (PRISMA) (25). The study was published in the Prospective Registry for Systematic Evaluation (PROSPERO) on February 20, 2024, under the registration number CRD42024507248.

### Data sources

This study searched eight databases from January 1, 2020, to January 1, 2024 [PubMed, Embase, Cochrane Central Register of Controlled Trials (CENTRAL), Web of Science (SCI), China Biomedical Database (CBM), China National Knowledge Infrastructure (CNKI), Wanfang Data Knowledge Service Platform and VIP Journal Integration Platform (VIP)], which it contains four English databases and four Chinese databases. The keywords used in the PubMed search included acupuncture, traditional Chinese medicine, leiomyomas, and randomized controlled trials. For specific search strategies, please refer to the hyperlink. PubMed retrieval type.txt.

### Study selection and data extraction

Two researchers (HW and MXJ) independently screened the retrieved literature, reviewed titles and abstracts, and excluded duplicate and irrelevant studies. Based on the inclusion and exclusion criteria, eligible studies were identified, and data were extracted and cross-checked. Ambiguities were resolved through discussion. If consensus could not be reached, a third researcher (CX) was consulted to make the final decision. All excluded studies were recorded. The experimental group received acupuncture (including needling, moxibustion, electroacupuncture, and catgut implantation at acupoints) in combination with traditional Chinese medicine, while the control group received only traditional Chinese medicine. The inclusion criteria were as follows: (a) Patients definitively diagnosed with uterine fibroids through clinical and ultrasound or gynecological examinations, with no restrictions on age, gender, or source of the case; (b) the experimental group was treated with acupuncture combined with traditional Chinese medicine, and the control group was treated with traditional Chinese medicine alone; (c) The study was a randomized controlled trial. The exclusion criteria were: (a) Subjects receiving treatments other than acupuncture and Chinese herbal medicine; (b) Duplicate publications; (c) Non-randomized controlled trials or studies with multiple control groups; (d) Reviews, systematic evaluations, animal studies, dissertations, case reports, theoretical investigations, conference abstracts, and literature not aligned with the study’s purpose; (e) Studies with poorly designed trials or mismatched methodologies; (f) Studies with apparent errors or omissions; (g) Studies with incorrect or unavailable data. (**Table 1**).

**Table 1:** Characteristics of the studies included in this systematic review (acupuncture + Chinese herbs vs. Chinese herbs).

NO.	Study, publication year (country)	No. of patients (O/A)	Age: mean ± SD or range (years)	Duration of uterine fibroids: mean ± SD or range (years)	Intervention	Control	Period of treatment	Side effects and adverse events	Type of outcomes
1	Huang Q, 2019 (27) (China)	I:30/30 C:30/30	I:42.19 ± 3.82 (27-51) C:42.73 ± 3.94 (28-52)	I:2.43 ± 0.31 C:2.52 ± 0.34	Acupuncture + Traditional Chinese medicine	Traditional Chinese medicine	3 months	NR	total clinical efficiency, the volume of uterine fibroids
2	Fu JY, 2014 (28) (China)	I:41/41 C:40/40	I:41.5 ± 8.2 (22-50) C:40.8 ± 7.9 (21-49)	I:3 months-6 years C:4 months-5.5 years	Acupuncture + Traditional Chinese medicine	Traditional Chinese medicine	6 months	NR	total clinical efficiency, E2, FSH, LH, P
3	Zhang Y, 2018 (29) (China)	I:35/35 C:33/33	I:38.4 ± 2.5 (31-45) C:38.5 ± 2.5 (31-47)	I:3.0 ± 0.6 C:3.1 ± 0.6	Acupuncture + Traditional Chinese medicine	Traditional Chinese medicine	3 months	NR	total clinical efficiency, the volume of uterine fibroids
4	Zhang X, 2015 (30) (China)	I:40/40 C:40/40	I:44.81 ± 5.66 (20-60) C:47.49 ± 4.36 (20-60)	NR	Acupuncture + Traditional Chinese medicine	Traditional Chinese medicine	3 months	NR	total clinical efficiency, the volume of uterine fibroids
5	Yang Z, 2019 (31) (China)	I:30/30 C:30/30	I:47.8 ± 5.1 (NR) C:48.2 ± 4.5 (NR)	I:1.5 ± 0.4 C:1.9 ± 0.5	Acupuncture + Traditional Chinese medicine	Traditional Chinese medicine	1.4 months	NR	total clinical efficiency, the volume of uterine fibroids, Clinical symptoms, Quality of life
6	Peng YS, 2010 (32) (China)	I:32/32 C:30/30	I:38.6 ± 7.1 (30-57) C:37.9 ± 6.8 (31-59)	NR	Acupuncture + Traditional Chinese medicine	Traditional Chinese medicine	2 months	NR	total clinical efficiency, the volume of uterine fibroids
7	Hu JP, 2018 (33) (China)	I:43/43 C:43/43	I:43.7 ± 3.2 (30-62) C:43.7 ± 3.2 (30-62)	NR	Acupuncture + Traditional Chinese medicine	Traditional Chinese medicine	2 months	NR	total clinical efficiency, the volume of uterine fibroids
8	Zhou QL, 2018 (34) (China)	I:40/40 C:40/40	I:37.69 ± 2.14 (23-51) C:35.88 ± 2.26 (19-54)	I:1.7 ± 0.27 C:1.6 ± 0.24	Acupuncture + Traditional Chinese medicine	Traditional Chinese medicine	3 months	NR	total clinical efficiency, the volume of uterine fibroids
9	Wan LX, 2013 (35) (China)	I:32/32 C:32/32	38.5(30-49)	NR	Acupuncture + Traditional Chinese medicine	Traditional Chinese medicine	3 months	NR	total clinical efficiency, the volume of uterine fibroids

1: estradio (E2); 2: follicle-stimulating hormone (FSH); 3: Luteinizing Hormone (LH); 4: Progesterone (P).

### Risk of bias assessment

**Figure 1:**
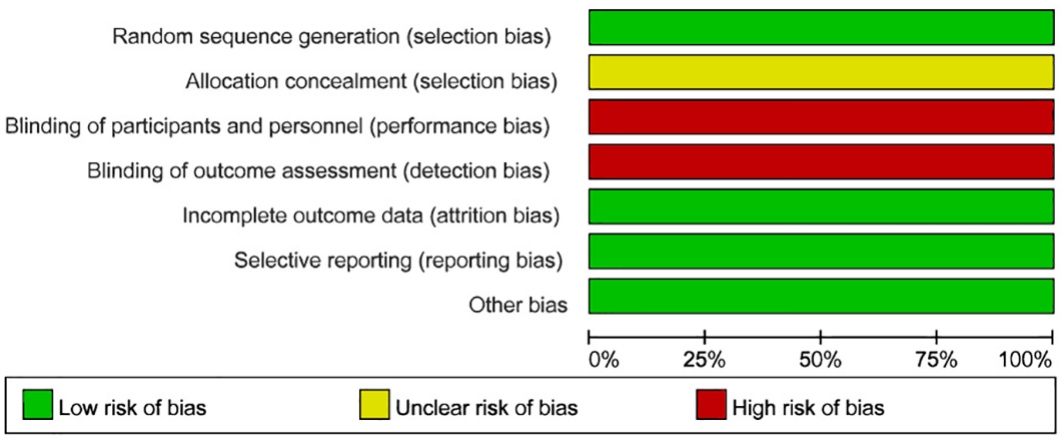
Risk of bias summary.

**Figure 2:**
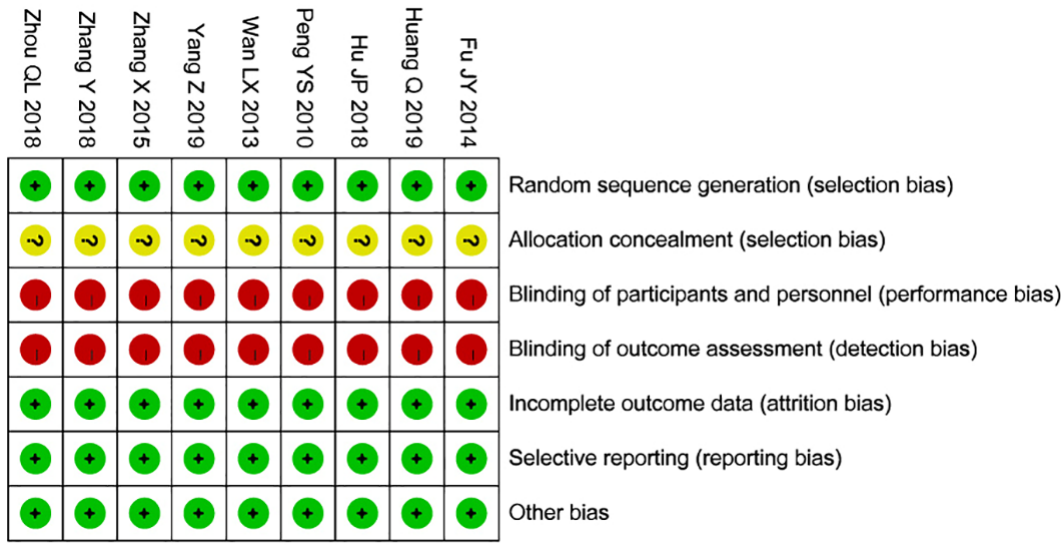
Risk of bias graph.

The Cochrane tool was used for the risk of bias assessment (26). This tool evaluates seven key aspects: 1) Baseline comparability—whether participants in different groups are similar in key characteristics (e.g., age, sex, disease severity); 2) Random allocation method—whether participants were randomly assigned, giving each participant an equal chance of being placed in any group; 3) Concealed allocation—whether the researcher and participant were unable to predict the group assignment prior to the allocation; 4) Blinding—whether the participant, researcher, or outcome assessor was unaware of the type of intervention the participant received; 5) Completeness of outcome data—whether outcome data for all participants were fully reported; 6) Selective reporting—whether the researcher selectively reported only certain outcomes (e.g., reporting only significant results while ignoring non-significant outcomes); 7) Other sources of bias—for example, potential conflicts of interest in the study’s funding, representativeness of participants, or unintended interventions or changes during the study’s implementation. The results of the risk of bias assessments were entered into Review Manager 5.4 statistical software, and the risk of bias for each of the seven aspects was categorized as “low risk,” “high risk,” or “unclear risk.”Two researchers (HW and MXJ) independently assessed these factors, and a third researcher (CX) was consulted to resolve any disagreements (**Figures 1**, **2**).

### Outcomes

Primary outcome measure: The degree of reduction in uterine fibroid size.

### Statistical analysis

The I² test and Q test were first applied to evaluate whether there was heterogeneity among the studies, and then an appropriate effect model was selected. When P > 0.05 and I² < 50%, it indicates no statistical heterogeneity among the effect sizes of the studies, and a fixed-effect model was used; When P ≤ 0.05 and I² ≥ 50%, it indicates statistical heterogeneity among the effect sizes, and a random-effects model was selected. If statistical heterogeneity was present, the potential sources of heterogeneity were explored, and sensitivity analysis was used to assess the stability of the results if necessary. Risk Ratio (RR) was used as the effect size for categorical data and expressed as 95% confidence intervals (CI), while Mean Difference (MD) or Standardized Mean Difference (SMD) was used for continuous data in the calculation of combined statistics. Results of the meta-analysis were represented using forest plots, funnel plots, and other graphical methods.

## Results

### Studies retrieved

**Figure 3:**
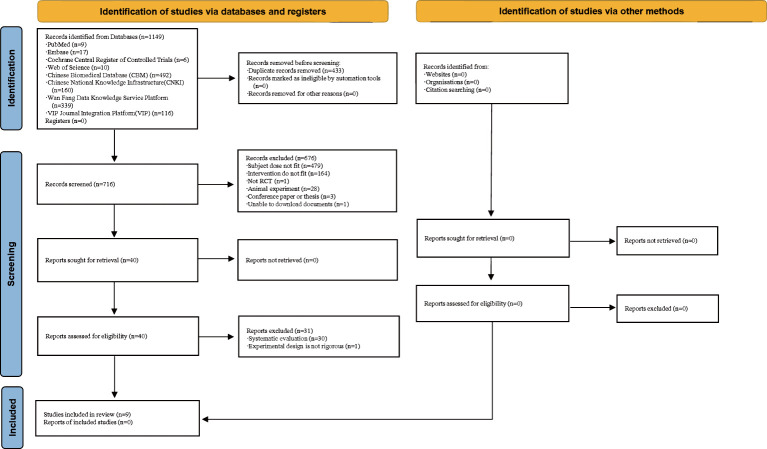
Flowchart of the study selection process.

A total of 1,149 relevant papers were initially identified, and after multiple rounds of screening, nine studies (27–35) met the inclusion criteria. These nine randomized controlled trials included 640 patients who received either acupuncture or acupuncture combined with Chinese herbs for the treatment of uterine fibroids (**Figure 3**).

### Quality of the evidence: summary of findings table

**Figure 4:**
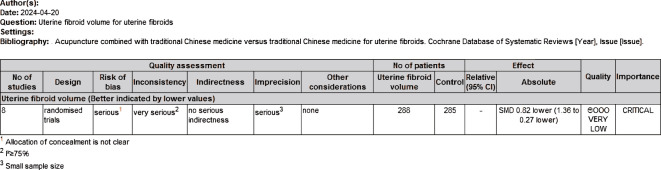
Quality of evidence assessment scale for uterine fibroid volume.

**Figure 5:**
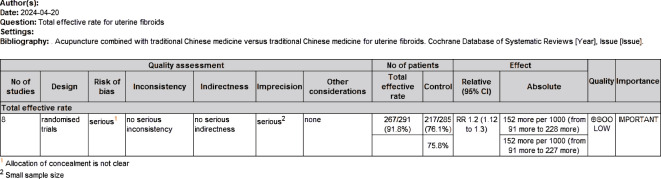
Quality of evidence assessment scale for total effective rate.

We created a “Summary of Results” table using the GRADE method to assess the quality of evidence for the outcome measures (size of uterine fibroids and overall clinical efficacy rate) in studies comparing acupuncture combined with Chinese herbs to Chinese herbs alone. The quality of evidence was evaluated using GRADE criteria, which include factors such as study limitations, inconsistency, indirectness, imprecision, and publication bias. Two investigators (HW and CTY) independently assessed the quality of the evidence (categorized as high, moderate, low, or very low) and resolved any differences through discussion. The results indicated that the quality of evidence for the reduction in uterine fibroid size was rated as “very low,” while the quality of evidence for the overall clinical efficacy rate was rated as “low” (**Figures 4**, **5**).

### Results of the risk of bias assessment using the Cochrane tool

The results of the Cochrane risk of bias assessment showed the following: 1) Baseline comparability was satisfied and judged as low risk of bias. 2) The studies by Huang Qing (27) and Yang Zhen (31) used randomized allocation with random numbers, and the study by Zhou Qinglian (34) used a completely randomized allocation method. The other studies stated that they were randomized controlled trials but did not describe the specific randomization methods; these were judged as low risk of bias. 3) All studies did not explicitly report on allocation concealment, so they were judged as having unclear risk of bias. 4) All studies were non-blinded due to the limitations of the interventions and were judged as high risk of bias. 5) The outcome data were complete, so they were judged as low risk of bias. 6) None of the studies selectively reported results, so they were judged as low risk of bias. 7) None of them had any other sources of bias, judged as low risk of bias. (**Table 2**).

**Table 2:** Cochrane risk of bias assessment.

NO.	Author	Publication year	Baseline comparability	Random allocation	Allocation concealment	Blinding	Integrity of results data	Selective reporting of findings	Other sources of bias
NO.1	Huang Q	2019	√	①	Inaccurate	Non-blind	√	×	×
NO.2	Fu JY	2014	√	②	Inaccurate	Non-blind	√	×	×
NO.3	Zhang Y	2018	√	②	Inaccurate	Non-blind	√	×	×
NO.4	Zhang X	2015	√	②	Inaccurate	Non-blind	√	×	×
NO.5	Yang Z	2019	√	①	Inaccurate	Non-blind	√	×	×
NO.6	Peng YS	2010	√	②	Inaccurate	Non-blind	√	×	×
NO.7	Hu JP	2018	√	②	Inaccurate	Non-blind	√	×	×
NO.8	Zhou QL	2018	√	③	Inaccurate	Non-blind	√	×	×
NO.9	Wan LX	2013	√	②	Inaccurate	Non-blind	√	×	×

1: Random number—①; Randomized control methods, but no specific random assignment method described—②; Totally random—③.

## Main results

### Size of uterine fibroids

**Figure 6:**
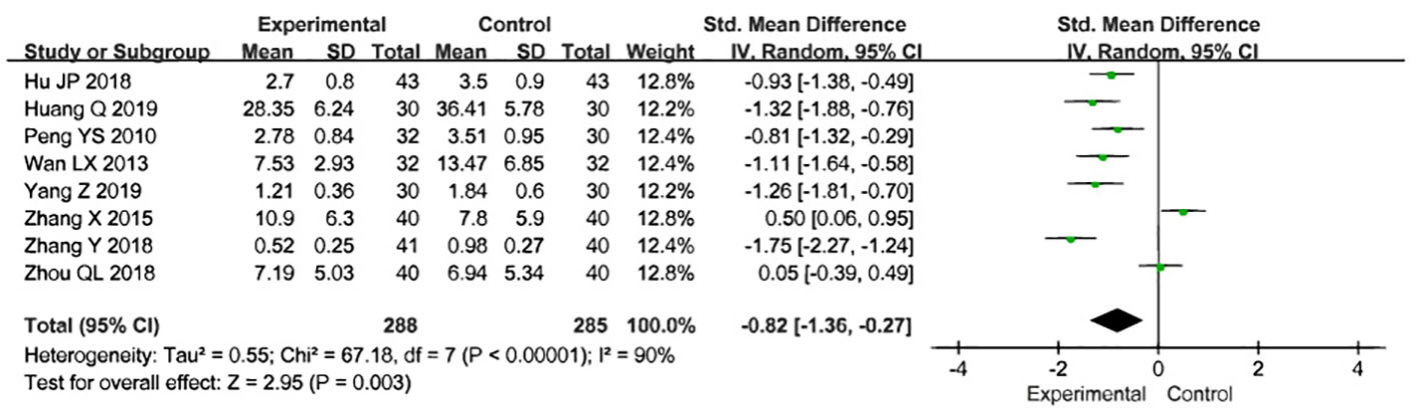
Meta analysis results of acupuncture combined with traditional Chinese medicine in the treatment of uterine fibroid volume reduction.

**Figure 7:**
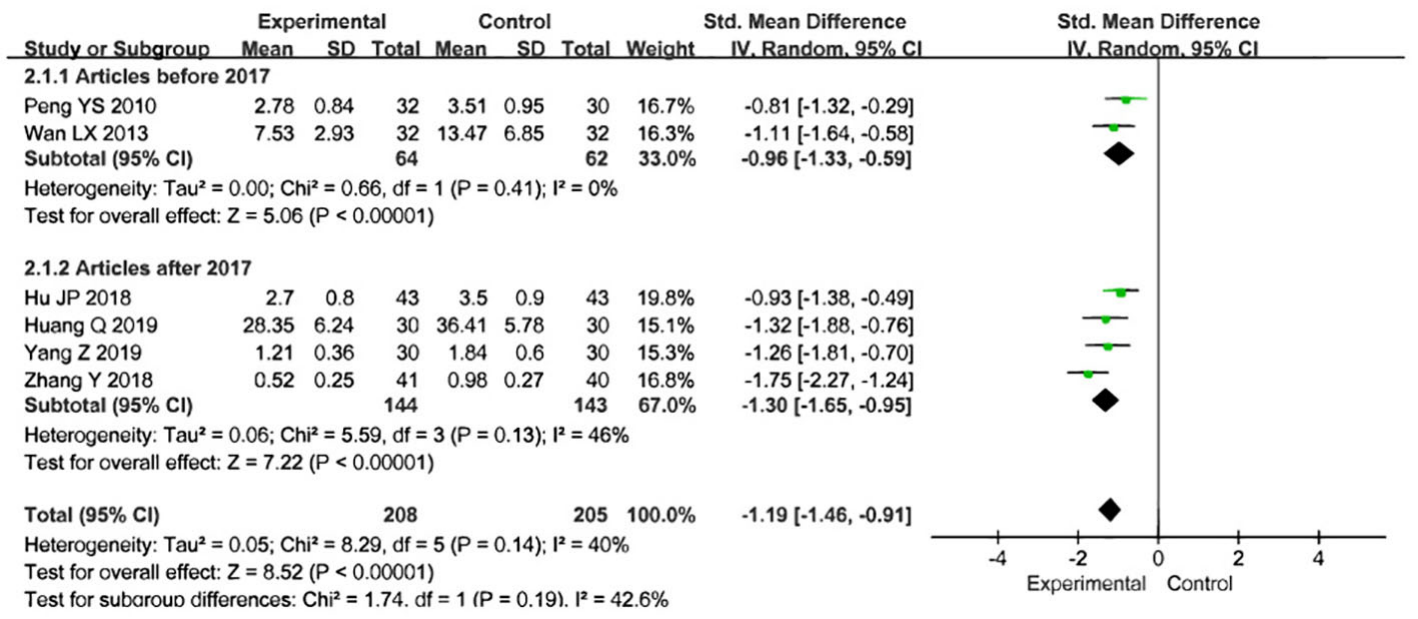
Subgroup analysis of uterine fibroid volume.

Eight studies used fibroid volume to assess the effectiveness of treatment, involving a total of 573 participants, with 288 in the treatment group and 285 in the control group. After treatment in both groups, fibroid volume was used as the joint effect measure. The heterogeneity test revealed substantial heterogeneity, so the random-effects model was selected for the meta-analysis [Z=2.95, P=0.003, SMD= -0.82,95%CI - 1.36 to -0.27, P<0.00001, I²=90%] (**Figure 6**), Sensitivity analyses were performed by excluding the studies by Zhang X (2015) and Zhou QL (2018), which resulted in P = 0.14 and I² = 40%. To further explore the cause of heterogeneity, we excluded the two aforementioned studies and conducted a subgroup analysis based on the publication year of the studies. The results showed P = 0.41 and I² = 0% for studies published before 2017, and P = 0.13 and I² = 46% for those published after 2017. We found that the heterogeneity for studies published before 2017 (I² = 0%) was significantly lower than the overall heterogeneity (I² = 40%). Therefore, the source of heterogeneity may be related to the publication year of the studies (**Figure 7**).

### The overall clinical efficacy rate

**Figure 8:**
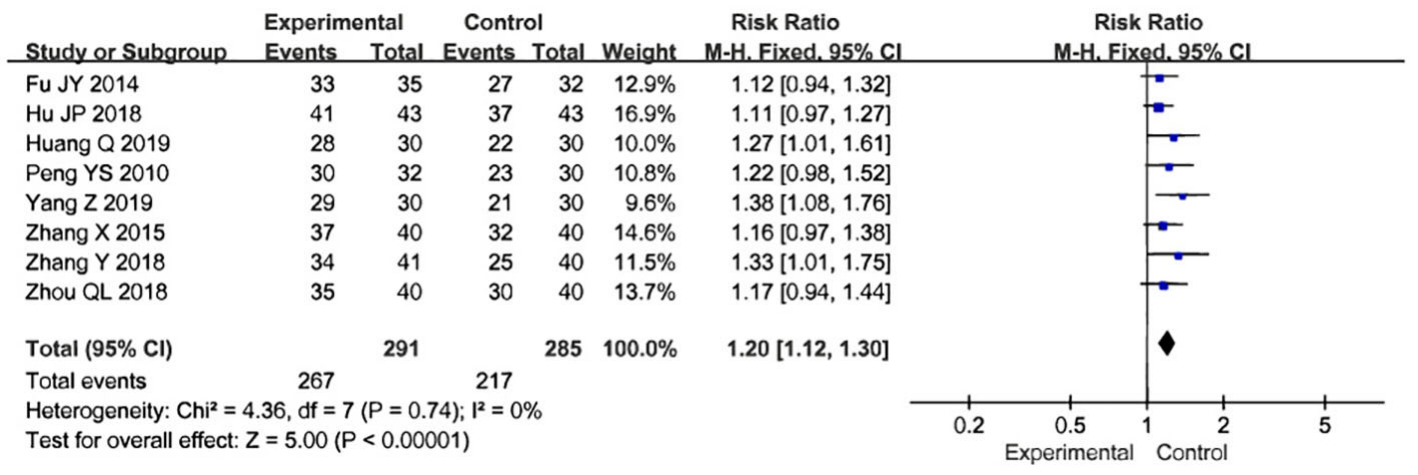
Forest map of total efficiency of acupuncture combined with traditional Chinese medicine in the treatment of uterine fibroids.

**Figure 9:**
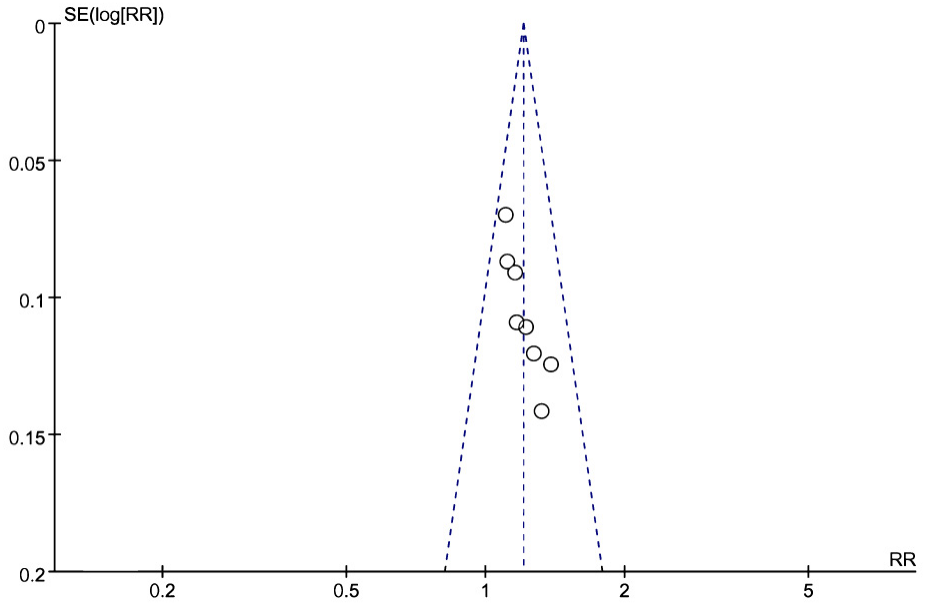
Funnel map of total effective rate of acupuncture combined with traditional Chinese medicine in the treatment of uterine fibroids.

Eight studies used the overall clinical efficacy rate to evaluate treatment outcomes. These studies included 576 participants, with 291 in the treatment group and 285 in the control group. A test for heterogeneity was conducted, revealing low heterogeneity and statistical significance, allowing for meta-analysis using a fixed-effects model [Z=5.00, P=0.74, relative risk(RR)1.20,95%CI 1.12 to 1.30, P<0.00001, I²=0%]. To ensure the accuracy and stability of the results, a sensitivity analysis was performed, which showed that none of the studies significantly affected the results of the meta-analysis. This indicates that the study had good stability and suggests that the overall treatment effect in the experimental group was better than that in the control group (**Figure 8**). A funnel plot was generated, which appeared symmetrical, suggesting no significant publication bias in the included studies. However, some data points were located on the lower right side of the funnel, which may indicate that the relative risk (RR) of some findings was higher than the average. While the funnel plot did not show obvious serious bias, this does not completely rule out the possibility of publication bias. Funnel plots provide only a preliminary visual assessment and cannot guarantee the absence of potential bias. For example, studies with non-significant results may not have been published, leading to a bias favoring studies with positive outcomes. Additionally, the shape of the funnel plot may be influenced by the sample size of the included studies. The relatively small number of studies included in the plot reduces its statistical power. (**Figure 9**).

### Other outcome indicators

Only a few studies mentioned uterine volume, hormone levels, clinical symptoms, quality of life, and adverse effects, but these will not be analyzed due to incomplete data.

## Discussion

### Summary of main results

The meta-analysis included data from nine randomized controlled trials and 640 participants to compare the efficacy and safety of acupuncture combined with Chinese herbs versus Chinese herbs alone in the treatment of uterine fibroids. The results showed that acupuncture combined with Chinese herbs significantly increased the overall clinical effectiveness for uterine fibroids compared with Chinese herbs alone [Z=5.00, P=0.74, relative risk(RR)1.20,95%CI 1.12 to 1.30, P<0.00001, I²=0%] (27–34), and also reduced the size of uterine fibroids [Z=2.95, P=0.003, SMD= -0.82,95%CI - 1.36 to -0.27, P<0.00001, I²=90%] (27, 29–35). Additionally, a few studies have shown that acupuncture combined with Chinese herbs can reduce uterine size (30), lower hormone levels (E2, FSH, LH, P) (28), and improve quality of life (31).

Subgroup analysis of uterine leiomyoma volume indicated that the source of heterogeneity was related to the publication date of the studies. Several factors may account for this. First, the increased heterogeneity between studies conducted before and after 2017 may be related to changes in diagnostic and treatment standards. In China, the diagnostic criteria and treatment protocols for uterine fibroids have evolved in recent years due to advances in medicine and updated guidelines. For example, starting in 2017, new diagnostic equipment and techniques, such as more precise ultrasound and MRI, were widely adopted, likely leading to more accurate measurements of fibroid volume. This suggests that studies conducted after 2017 reported more accurate data on fibroid volume, whereas pre-2017 studies relied on more basic measurement techniques, resulting in a wider range of data and reducing comparability. Second, earlier treatments primarily involved surgery and traditional Chinese medicine, while newer combination therapies, such as acupuncture, moxibustion, and acupoint catgut embedding, have gradually become more prevalent in clinical studies over time. The introduction of these newer therapies may have introduced additional variables between studies. For instance, acupoint catgut embedding combined with Chinese herbal medicine significantly reduced leiomyoma size, whereas Chinese herbal medicine alone had a relatively weaker effect. These differences in treatment regimens likely contributed to increased heterogeneity in outcomes, particularly as newer therapies became more commonly studied after 2017. Finally, studies conducted before 2017 may have employed simpler statistical methods, while more recent studies, benefiting from advancements in study design and statistical analysis—such as the wider use of random-effects models—are better able to capture data variability. As a result, post-2017 studies may display greater heterogeneity due to the use of more sophisticated analytical methods.

These findings are consistent with previous systematic reviews and randomized controlled trials. Additionally, several studies have demonstrated that acupuncture, either alone or in combination with traditional Chinese medicine, can more effectively improve various clinical symptoms in patients with uterine fibroids. These studies further validate our results.

Secondly, acupuncture works to reduce the size of fibroids by selecting appropriate acupuncture points based on the patient’s specific symptoms. While acupuncture therapy does not directly cause fibroids to disappear, it can help shrink them by regulating the body’s endocrine system, improving the circulation of qi and blood, and exerting an inhibitory effect on fibroid growth.

Clinical trials have shown that acupuncture has a positive effect on endometriosis, significantly reducing pain levels, shortening the duration of pain, relieving anxiety and depression, and improving patients’ quality of life, as indicated by the Visual Analog Scale (VAS) score. However, its efficacy diminishes once treatment is stopped (36). Additionally, acupuncture has been shown to reduce the size of ectopic cysts, lower serum carbohydrate antigen 125 (CA125) levels, regulate serum sex hormones, improve ovarian reserve function, increase pregnancy rates, and reduce recurrence rates and adverse effects. The mechanisms of acupuncture primarily involve the inhibition of angiogenesis, suppression of estrogen and its receptors, regulation of immune function, and pain relief (37). Animal experiments have demonstrated that electroacupuncture promotes the transition of the uterus to the active phase in late-pregnant rats by altering hormone levels in serum and uterine tissues, and it enhances coordinated uterine contractions by increasing the expression of uterine gap junction proteins (38).

Overall, these findings suggest that acupuncture provides more effective treatment for uterine diseases compared to herbal medicine alone.

### Overall completeness and applicability of evidence

None of the nine studies reported follow-up or withdrawal data, making it difficult to assess the long-term effects of treatment and relapse rates. Additionally, no violations of assigned treatments were noted. None of the studies specified whether the trial investigator and outcome assessor were the same person, which introduces potential bias. Bias may arise when the trial investigator also assesses participants’ outcomes. Furthermore, only one study reported adverse effects (31). Although the benefits of acupuncture appear to outweigh its drawbacks, we are not entirely confident about its safety. The total sample size of 640 patients across nine trials results in a small average sample size per trial, which may have led to a lack of statistical power and an increased likelihood of false-negative results. Even if the treatment effect is significant, the study may not detect it due to insufficient sample size. Trials with small sample sizes are more susceptible to the influence of individual data points, leading to unstable results. For example, an individual patient’s response may disproportionately impact the overall findings, making conclusions vulnerable to being skewed by outliers. Additionally, rare adverse reactions may go undetected due to the limited statistical data. The limited number of randomized controlled trials and participants means that the available evidence on the effectiveness of acupuncture combined with Chinese herbs for treating uterine fibroids remains incomplete. Future research should focus on conducting multicenter randomized controlled trials to increase sample sizes and improve statistical power. Multicenter studies can cover broader regions and populations, enhancing the generalizability of the results. For instance, recruiting patients from different racial and geographic backgrounds—such as populations in Asia, Europe, and Africa—could help validate the treatment’s applicability across diverse groups. International collaborations could also expand patient recruitment and facilitate resource sharing. Collaborating with hospitals and research institutions in other countries would help validate the global efficacy of acupuncture and herbal medicine for uterine fibroids.

### Quality of the evidence

Overall, this meta-analysis had a low risk of bias (**Figures 1**, **2**), but was rated as high risk in terms of blinding, as all trials were unblinded. This lack of blinding may lead to several issues: (1) When researchers know which treatment participants received, they may unintentionally influence their evaluation or recording of the results; (2) When participants know they are receiving a particular treatment, their expectations and attitudes may influence the results. This phenomenon is known as the placebo effect or the nocebo effect (anti-placebo effect); (3) Participants’ compliance may be affected when they are aware of receiving either a new drug or a control treatment. If participants know they are assigned to the control group, they may lose motivation to continue participating, leading to decreased adherence or even withdrawal. This may affect data integrity and the accuracy of the analysis; (4) In some cases, non-blinded trials are designed for ethical reasons, such as ensuring participants are fully informed. However, researchers, aware of the treatment allocation, may unintentionally alter their care or treatment of participants, affecting the objectivity of the trial; (5) Bias may lead to overestimation or underestimation of effect sizes. It becomes difficult to discern whether the observed effects are genuine results of the treatment or artifacts of the researchers’ and participants’ expectations. While statistical models can adjust for potential bias, they cannot entirely eliminate its effects. Moreover, none of the studies mentioned whether allocation concealment was implemented. The source of bias may also be related to participant characteristics (e.g., age, diagnostic criteria, severity of uterine fibroids), the method of acupuncture (e.g., needling, electroacupuncture, moxibustion, or acupoint catgut embedding), the selection and dosage of Chinese herbs, specific symptoms, and the course of treatment. Some studies also had limited reporting due to small sample sizes and differences in interventions. Therefore, the results of this meta-analysis should be interpreted with caution.

### Potential biases in the review process

We conducted an extensive search across multiple databases to reduce the likelihood of missing relevant studies. We also recognized the potential for publication bias, as this systematic review did not include trials with negative results. However, all the randomized controlled trials included in this study were conducted in China, with the study population primarily consisting of Chinese women. These participants were treated with acupuncture and herbal medicine, both of which are traditional Chinese therapies. This geographic and demographic limitation may affect the generalizability of the findings, particularly when applying them to non-Asian populations. Different ethnic groups and regions may exhibit significant variations in genetics, environment, lifestyle, and disease manifestation. As a result, the applicability and effectiveness of acupuncture combined with Chinese herbal treatments for uterine fibroids in non-Asian populations remain uncertain, necessitating further research across diverse regions and ethnicities to validate the broader relevance of these findings. Despite our efforts to search both Chinese and English databases, all the eligible studies were conducted in China, and the trial reports were brief, with some critical information either unclear or missing. Additionally, we were unable to obtain further clarification from the trial investigators despite attempts via email and phone. These factors may introduce bias into the current review.

### The actual contribution and impact of clinical practice

The contribution of this meta-analysis to clinical practice lies in its provision of systematic, quantitative evidence supporting the effectiveness of acupuncture combined with Chinese herbs in treating uterine fibroids. Compared with Chinese herbs alone, the combination of acupuncture and Chinese herbs enhanced the overall treatment effectiveness and significantly reduced the size of uterine fibroids. This finding serves as an important reference for physicians when selecting appropriate treatment options in clinical settings.

First, for clinicians, integrating acupuncture with Chinese herbs offers a more comprehensive treatment option for patients with uterine fibroids. Based on the results of this study, clinicians can consider acupuncture combined with herbal medicine as a safe and effective non-invasive treatment option for patients who prefer to avoid surgery or are not eligible for surgical interventions.

Secondly, for patients with large fibroids or severe symptoms, acupuncture combined with Chinese herbs can help reduce the size of fibroids by regulating the endocrine system and improving blood circulation, thereby alleviating associated symptoms and enhancing overall quality of life. This approach allows the therapy to play an active role in symptom management and long-term treatment.

In conclusion, the findings of this study provide a strong scientific basis for the integration of Chinese and Western medicine in the treatment of uterine diseases. Specifically, for uterine fibroids—a chronic condition—acupuncture combined with Chinese herbs offers a more comprehensive therapeutic option, underscoring the importance of individualized treatment. Selecting the appropriate acupoints and herbal formulas based on clinical symptoms can further improve the precision and effectiveness of treatment.

Despite the positive findings of this study regarding the effectiveness of acupuncture, clinicians should remain mindful of its limitations, such as the small sample size and insufficient follow-up data. Therefore, in clinical practice, it is recommended that patient symptom changes be regularly monitored when incorporating acupuncture into treatment, and that other conventional therapies be combined to maximize efficacy. Additionally, more high-quality clinical trials with long-term follow-up are needed to strengthen the reliability and broader applicability of the evidence.

## Conclusion

### Implications for practice

We cannot rule out the possibility of clinically relevant differences between acupuncture combined with Chinese herbs and Chinese herbs alone in terms of overall clinical efficacy, reduction in the size of uterine fibroids, levels of E2, FSH, LH, and P, as well as improvements in clinical symptoms and quality of life. Compared with Chinese herbs alone, acupuncture combined with Chinese herbs was found to improve overall clinical efficacy, reduce fibroid size, and positively affect hormone levels (E2, FSH, LH, P), clinical symptoms, and quality of life. However, due to the low quality of evidence in this field and the limited number of clinical trials available, we could only conclude that acupuncture combined with Chinese herbs appears more effective than Chinese herbs alone in treating uterine fibroids.

### Implications for research

It is hoped that acupuncture combined with Chinese herbs will improve the overall clinical outcomes for patients with uterine fibroids. Under unified diagnostic criteria, a standardized set of acupoints and stimulation methods should be used, and the control group should receive the same herbal treatment as the acupuncture group.

## Data Availability

The original contributions presented in the study are included in the article/supplementary material. Further inquiries can be directed to the corresponding author.
